# The Application of Pollen as a Functional Food and Feed Ingredient—The Present and Perspectives

**DOI:** 10.3390/biom10010084

**Published:** 2020-01-05

**Authors:** Aleksandar Ž. Kostić, Danijel D. Milinčić, Miroljub B. Barać, Mohammad Ali Shariati, Živoslav Lj. Tešić, Mirjana B. Pešić

**Affiliations:** 1 Faculty of Agriculture, Chemistry and Biochemistry, University of Belgrade, Nemanjina 6, 11080 Belgrade, Serbia; 2 Kazakh Research Institute of Processing and Food Industry (Semey Branch), Semey 071410, Kazakhstan; 3 Laboratory of Biocontrol and Antimicrobial Resistance, Orel State University Named After I.S. Turgenev, 302026 Orel, Russia; 4 Faculty of Chemistry, Analytical Chemistry, University of Belgrade, Studentski Trg 12-16, 11000 Belgrade, Serbia

**Keywords:** bee-collected pollen, floral pollen, feed ingredient, food ingredient, pollen-based food products, anti-nutrients

## Abstract

Pollen is recognized as an excellent dietary supplement for human nutrition, which is why it can be found in different forms on the market (granules, capsules, tablets, pellets, and powders). But, the digestibility of pollen’s nutrients is strongly affected by the presence of a pollen shell, which can decrease the bioavailability of nutrients by 50% and more. Since consumers have become more aware of the benefits of a healthy diet and the necessity to improve pollen digestibility, different pollen-based functional food products have been developed and extensive studies were done to estimate the beneficial effects of pollen-based feed on animal growth, health, and rigor mortise stage. Considering the positive effects of pollen nutrients and phytometabolites on human and animal health, the aim of this paper was to give an overview of recent achievements in the application of pollen in the formulation of functional food and animal diets. Special attention was paid to the effects of pollen’s addition on the nutritional, functional, techno-functional, and sensory properties of the new formulated food products. Anti-nutritional properties of pollen were also discussed. This review points out the benefits of pollen addition to food and feed and the possible directions in the further development of functional food and feed for the wellbeing of everyone.

## 1. Introduction

Although the term pollen was established in the 17th century from the Latin word which defines fine powder (flour), this plant material has been known as “food” for centuries [1]. For all Apidae insects, and for some which do not belong to this genus, pollen is the main food source during development. In this sense, worker bees use pollen for royal jelly production (basic food for the larval queen and worker larvae) or directly as food due to the great amount of proteins and lipids present in it [2]. As it immensely benefits the insects’ diet, pollen has become an important component of animal feed, above all, as an exceptional source of proteins and a good natural substitute for antibiotics [3]. Regarding pollen as food for humans, the first review was probably presented in the 70s; the authors [2] had reviewed the currently available data and concluded that for some athletes from different countries pollen as a dietary supplement was an important component for weight improvement and for the prevention of some respiratory infections. In addition, they emphasized the fact of the longevity of some residents of Ukraine and Russia which can be linked to their regular consumption of honey enriched with pollen [2]. Since then, a significant “breakthrough” about the application of pollen as a dietary supplement has been made and the number of available research articles has drastically increased.

**Figure 1:**
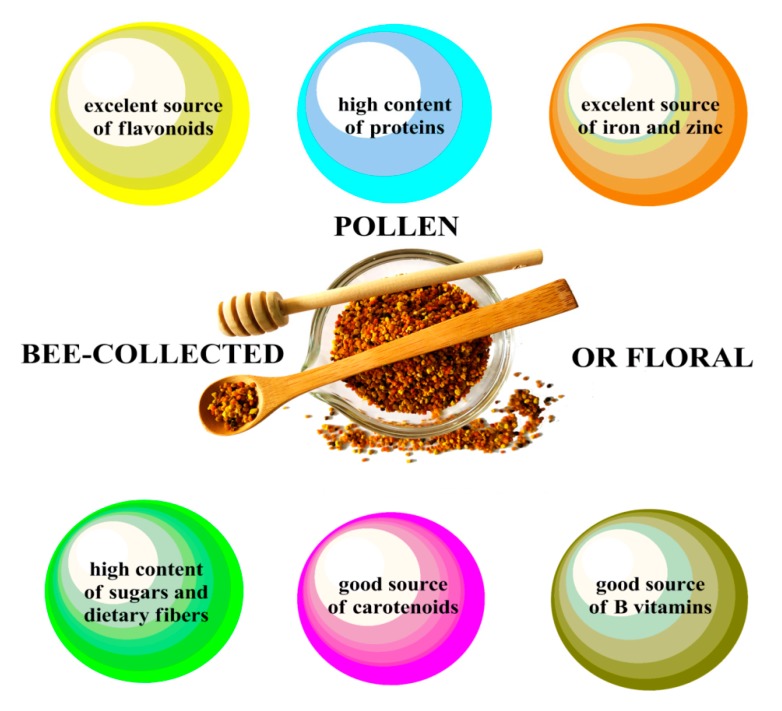
Pollen as a source of important food components.

Nowadays, pollen is often recognized as the “only perfectly complete food” [4], and “the world’s best food product” [5]. The dominant presence and high content of carbohydrates, proteins, and lipids highlights pollen as an ideal natural supplement that provides energy; it has good nutritional value, regulates certain biochemical functions, and strengthens the body’s immune and physiological systems [4,6]. Furthermore, pollen is a rich source of different important compounds (Figure 1)—vitamins (with the prevalence of group-B vitamins) [7,8], carotenoids (such as lutein, β-cryptoxanthin, and β-caroten) [9], minerals [10,11,12], and polyphenols [13,14,15], which makes it attractive for use in the diets of children and adults suffering from certain avitaminoses and loss of appetite.

Due to the diversity of active natural metabolites, especially vitamins, carotenoids, and polyphenols, pollen has a significant biological activity, expressed as the antioxidant, antibacterial, and anti-carcinogenic activity and the hepatoprotective, and the cardioprotective effect [6]. Furthermore, pollen is recognized as a good tool for the therapeutic treatment of several different nonallergic diseases [7]. It was reported that the flower pollen extracts can be used as a complementary remedy for the treatment of the benign prostatic hyperplasia, chronic prostatitis, and vasomotor symptoms in women, but its clinical efficacy should be further tested [7]. The most important pollen compounds which are believed to possess the most prominent pharmacological activity are essential fatty acids, phospholipids, phytosterols, flavonoids, and phenolic acids [6,7].

Thus, various pollen products can be found on the market in the form of granules, capsules, tablets, pellets, and powders [6]. The recommended daily dose of pollen consumption for an adult should range from 20 to 40 g. However, fresh or dried grains of pollen frequently have a hard shell (intine and exine) that can significantly affect the penetration of the digestive enzymes into the pollen pellets, and thus the absorption of important nutrients by the human digestive tract [5]. It was reported that an average degree of digestibility of carbohydrates was 4% or 53% for proteins depending on the origin of pollen [16]. Therefore, in order to increase the digestibility and the functionality, the pollen grains should be ground and dissolved in warm water, whereby the accessibility of nutrients increases to 60–80% [5].

In the literature, numerous review papers focus on a detailed overview of the pollen nutrients (carbohydrates, protein, amino acids, lipids, and minerals), their phytometabolites (carotenoids, phenols, flavonoids, and vitamins), and their positive effects on human health and possible therapeutic properties [5,6,17,18]. Recently, due to the increased awareness of consumers that the consumption of functional foods can improve their health, pollen began to be considered as a functional food and feed ingredient. So far, a number of fermented pollen-based food products have been developed [19,20,21,22,23,24,25,26,27,28,29,30,31,32,33,34,35,36,37,38,39], including bee-collected pollen-based baking [40,41], and confectionery [42,43], juice [44], and meat [45,46,47] food products; the dietary use of bee pollen as a feed additive for livestock and poultry revealed that bee pollen improves growth, reproductive, and immune performance of animals [3,48,49,50,51].

Despite all the benefits that can be obtained from pollen as a functional food ingredient, it should be mentioned that pollen can possess anti-nutritional compounds, such as allergens, pyrrolizidine alkaloids, toxic and potentially toxic elements, and mycotoxins [10,11,52,53]. For a specific group of consumers, some allergic reactions will be expressed if they are sensitive to the presence of pollen. According to literature, pollen-related food allergy (PRFA) is commonly accompanied by the rapid development of oral allergy symptoms after food ingestion, with spontaneous resolution within 10–30 min [52], and the vast majority of potentially allergic people tolerate these foods after heating by boiling, baking, or cooking [52], but PRFA should not be overlooked. The occurrence of hepatotoxic pyrrolizidine alkaloids was registered in the bee-collected pollen from *Echium*, *Eupatorium*, and *Senecio* plants that synthetized these compounds. [53,54]. The presence of mycotoxins, mycotoxin producing fungi, and toxic and potentially toxic elements is mostly due to inadequate handling of the pollen grains. [10,55,56].

Considering the significance of pollen and the benefits of its use for human and animal health, the aim of this paper was to give and overview of recent achievements in the application of pollen in the formulation of the functional food and the animal diet. Special attention was paid to the effects of pollen addition on the nutritional, functional, techno-functional, and sensory properties of the new formulated food products and to the effects on animal growth, health, and rigor mortis stage. Anti-nutritional properties of pollen were also discussed. To the best of our knowledge this is the first report which summarizes all these findings.

## 2. The Application of Pollen as a Functional Food Ingredient

For the application of pollen as a functional food ingredient, it is extremely important to study and understand the adequate way(s) for storing and preservation of pollen in order to keep all the nutrients. It is well known that pollen’s viability and germination ability decrease during inadequate storage and that they can be affected by several factors, such as humidity, temperature, gas atmosphere, and oxygen pressure [2]. Similarly, there are different factors (microbiological, chemical, physical, and mechanical) that may affect the nutrient/bioactive components’ contents in food/pollen before it is used [57]. For these reasons, a suitable preservation process should be applied, and regular quality control must be conducted throughout the whole time.

### 2.1. The Effect of Drying Techniques and Storage Conditions on the Quality of Bee-Collected Pollen

Freshly collected pollen contains high levels of moisture (usually 20%–30%) and increased water activity (*a_w_*) which is conducive to the rapid development of various microorganisms and chemical and enzymatic reactions; thus, reducing the shelf life and the potential for pollen utilization [5,58,59]. According to Gonzalez et al. [60], water activity of pollen that is ready for consumption should be in the range of 0.261–0.280, but the drying process and storage conditions influence the quality of bee-collected pollen—both in terms of the nutritional properties and the bioactive compounds [61,62,63,64,65,66].

#### 2.1.1. The Influence of Drying Techniques on the Quality of Bee-Collected Pollen

For the preservation of freshly collected pollen, different drying techniques—hot-air drying, freeze-drying, microwave drying, vacuum drying, and microwave-assisted vacuum drying, are used [58,61,62,64,67,68,69] (freeze-drying and hot-air drying are most commonly applied [58,61,62,67,68,69]). Drying the pollen with hot air is recommended at the temperature of 40 °C, while at higher temperatures the physicochemical properties, morphological structures, and organoleptic characteristics of pollen are disturbed [62]. For example, drying the pollen samples at 60 °C reduced the amounts of protein and vitamin C by 43.7% and 31.5%, respectively, changed the pollen colour (ΔE value 9.19 ± 2.11), and significantly decreased the sensory attributes (visual appearance, colour, and odour) of the dried pollen compared to the fresh one [62]. Furthermore, Collin et al. [66] pointed out that only the drying treatment of pollen at 30 °C, or for a short time at 40 °C, can be effective at avoiding the synthesis of dimethyl sulphide (pollen is rich in 5′-methylmethionine), undesirable aldehydes, and furan compounds, and preventing the loss of the desirable monoterpenic compounds.

However, freeze-drying is defined as an ideal method for treating the commercially collected pollen, since the individual compounds present in pollen are kept within the range required to standardize the commercial pollen as a product available in markets [58,61,63,69,70]. Comparing drying treatments, the lyophilization of the pollen samples has proven to be better for preserving total polyphenols, total flavonoids, and antioxidant activity compared to air drying at 42 °C [63]. Namely, Dias et al. [63] reported that the concentration of total polyphenols ranged from 16.67 ± 0. to 47.20 ± 1.99 mg GAE/g of the lyophilized pollen, whereas for the air-dried bee pollen values were between 12.75 ± 0.25 and 35.05 ± 0.05 mg GAE/g pollen, in the same samples. The total flavonoid content was higher by 24.6% to 41.9% in the lyophilized pollen samples than in the air-dried ones, although the total flavonoid content in the bee pollens of *Rubus* spp. and *Cytisus* spp. was not influenced by the preservation techniques. According to these findings, the lyophilized pollen samples showed lower EC_50_ values in the BCB assay (ranging from 0.71 ± 0.04 to 3.54 ± 0.09 for the lyophilized samples and from 2.16 ± 0.03 to 5.01 ± 0.15 for the air-dried samples), and a higher antioxidant activity estimated by the ORAC assay (from 149.94 ± 6.75 to 284.69 ± 13.69 mol eq. Trolox/g pollen for the lyophilized samples and from 241.40 ± 11.41 to 403.30 ± 14.36 mol eq. Trolox/g pollen for the air-dried samples). Moreover, Domínguez-Valhondo et al. [61] reported that the freeze-dried pollen had similar attributes to the fresh one (the amounts of fructose, glucose, sucrose, dietary fibre, and protein were unchanged, whereas the lipid content increased), but the content of free amino acids and bioactive compounds decreased during the drying processes, more severely in the hot-air-dried samples than in the lyophilized ones. The amounts of malvidin, lutein, and proline were specially affected. Although the lipid composition of the bee-collected pollen was not affected by freeze-drying, the tocopherol composition significantly changed after the freeze-drying treatment, showing the reduction of α-, δ- and γ-tocopherol content for 60%, 75%, and 90%, respectively [70]. The drying techniques based on the microwave and/or vacuum treatments can be used to process pollen samples; however, these techniques significantly affect the diastasis activity and increase the hydroxymethylfurfural (HMF) levels [64]. On the other hand, Canale et al. [65] emphasized that microwave drying is an ideal drying technique for the preservation of the bioactive compounds of fresh pollen, such as flavonoids (above all rutin) and amino acids, but it almost completely eliminates the content of tocopherols [70]. In addition, the pollen drying conditions are often closely related to the botanical origin and properties of pollen, as reported by Thakur and Nanda [71] who provided an example of a rapid and efficient drying method for the highly porous coriander pollen. Domínguez-Valhondo et al. [61] also concluded that the floral origin (polyfloral-type versus monofloral-type) had a slight influence on the nutritional properties of pollen, but significantly influenced the abundance of bioactive compounds. Several countries, such as Switzerland, Argentina, Brazil, and Poland, defined the microbiological and quality criteria that the dried pollen must meet in order to be used for human consumption [5,70].

#### 2.1.2. The Influence of Storage Conditions on the Quality of Bee-Collected Pollen

The quality of pollen and its formulas depend on the stages of its production process such as cleaning, packaging, transportation, and storage time [5,58]. According to Thakur and Nanda [71], the bee-collected pollen size distribution data are necessary to design the equipment for cleaning, classifying, and separating, while the physical properties such as 1000-pollen weight, bulk density, true density, and porosity are very important for the design of the equipment related to drying, storage, and transportation of pollen. Furthermore, it was suggested that the quality of pollen, its nutritional and functional value, decline with time [8,72], while Žilić et al. [73] highlighted the occurrence of Maillard reactions and phenolic oxidation in the stored and heat-treated floral maize pollen. De Arruda et al. [8] examined the stability of B-complex vitamins and their vitamers (B_1_, B_2_, B_6_, and PP) in dried pollen samples over one year of storage under different conditions (room temperature with and without the light and the freezer) and indicated that their concentrations decreased with storage time and storage conditions, except for the vitamin B_1_ whose concentration was unchanged. A similar conclusion was made by Melo and Almeida-Muradian [72] in their report, where they suggested that the concentrations of vitamins C, E, and β-carotene decreased during storage (average losses of vitamins C, E, and β-carotene after one year of storage at room temperature protected from light were 50%, 15%, and 59%–76%, respectively); however, they emphasized the efficiency of pollen storage in the freezer (average losses of vitamins C, E, and β-carotene after one year of storage were 26%, 13%, and 12%, respectively). Decrease in the concentration of polyphenols over time in the bee pollen extracts was also observed in relation to storage conditions (reduction between 19.9% and 57.7% depending on the type of extracts) [74], while the loss of the nutritional value and the emergence of Millard reactions which formed during the inappropriate storage of pollen and heat treatment were associated with high amounts of protein, glucose, and fructose [73,75].

It is worth mentioning that the addition of bee-collected pollen to honey in different ratios may modify *a_w_* value of the bee-collected pollen-honey mixture during 90 days of storage [59]. Namely, it was reported that depending on the type of pollen, *a_w_* value of the mixture can be higher or lower than each component of the corresponding mixtures. Additionally, the formulation of the “honey-bee-collected pollen mix” possesses significant antinociceptive, anti-inflammatory, and antilipidperoxidant activities orally administrated to mice at 500 mg/kg. [76].

The processing and storage of pollen faces numerous challenges due to the poor stability of valuable pollen compounds. These difficulties could be solved relatively easily by the application of encapsulation technology. It was demonstrated that micro and nano-encapsulation of sensitive compounds such as polyphenols can prevent their degradation due to light, oxygen, pH, or heat treatments, providing stability during processing and storage [77,78].

### 2.2. Fermented Bee-Collected and Floral Pollen-Based Products

The application of fermentation in food production has been known throughout the whole of human history. Therefore, many products that are dependent on the fermentation process, such as bread, cheese, yogurt, wine, soy sauce, and fermented sausages, are of great importance for human nutrition [79]. Food preservation by fermentation provides extended shelf life, and microbiological safety; it improves digestibility and nutritional properties of some foods; it participates in the formation of textures and unique flavours; and in some cases, fermented foods may have probiotic properties [79,80]. Pollen has always been in the field of interest for food scientists, but recently a large focus has been put on providing the final products with improved characteristics, and a special part of the research is based on the fermentation as a tool for production of the bee-collected and the floral pollen-based products. More and more products are being obtained by fermentation of pollen or other raw materials to which pollen has been added. The presented studies (details are given in Table 1) define several important observations regarding the influence of bee-collected/floral pollen on the quality of final products:

- The fermentation of pollen with kombucha/SCOBY consortium can significantly improve the bioavailability of the bioactive compounds present in pollen, which leads to the formation of kombucha health-related components and the formation of a product exhibiting a moderate antitumor effect on Caco-2 cells. Following the course of pollen fermentation by kombucha/SCOBY consortium, Uțoiu et al. [19] showed an increased release of polyphenols (from 12.73 to about 34 mg/L) and flavonoids (from about 2.5 to about 5 mg/L) from pollen into the liquid phase of the fermented product, and the additional accumulation of organic acids, primarily hydroxy acids (citric, gluconic, and lactic acids), and short-chain fatty acids (acetic, propionic, and butyric acids). Furthermore, the increased antioxidant capacity of the fermented product was recorded by DPPH (from 1.35 ± 0.1 to 4.91 ± 0.11 Inhibition Grade%/mL) and TEAC (from 8.83 ± 0.17 to 22.95 ± 0.77 µg Trolox/mL) assays.
- The addition of pollen (in the range from 10 to 50 mg/L) during the production of honey wine increased the ethanol content (from 11.74% ± 0.06% to 12.39% ± 0.12% *v*/*v*), which may be directly related to the application of pollen as a fermentation activator [20]. Moreover, the content of the aromatic components proportionally increased with the concentration of pollen (the content of isoamyl alcohol ranged from 248.78 ± 2.29 to 317.60 ± 2.93 and esters from 639.64 ± 122.80 to 1722.40 ± 330.69), while the content of organic acids was variable.
- Similarly to the previous research, the addition of pollen in the range from 0.1 to 20 g/L during the production of Palomini and Riesling wines positively affected the fermentation process. The content of volatile compounds such as terpenes, esters, and aldehydes increased, while the content of higher alcohols, C6 alcohols, and acids was variable. The aromatic volatile compounds that gave the floral and fruity tones and the ideal sensory characteristics of these wines were observed when the applied pollen doses were below 1 g/L [22].
- Bee-collected pollen-enriched yogurts show improved antioxidant capacity, an increase in polyphenolic content, and significantly improved sensory properties (influenced by the botanical origin of pollen) compared to the conventional yogurt [24,27].
- The addition of pollen has a positive effect on the textural and techno-functional properties of yogurt, such as increased gel strength and decreased syneresis [25,26,29].
- The sensory properties of pollen-enriched yogurt often depend on the origin of pollen and its concentration (0.5; 1.0% and 1.5%). For example, according to Khider et al. [29], maize pollen supplementation gives a nutty flavour and an enhancement in the texture of the yogurt which can be related to high polysaccharide content of this pollen. The clover pollen gives a sweet taste, while the date palm pollen gives a bean-like flavour to the yogurt. Encouraged by the antimicrobial and antioxidant results, and the ability of pollen to inhibit lipid peroxidation, with the aim of extending the shelf life of yogurt, Khider et al. [29] confirmed the preserving properties of the Egyptian maize and the clover bee pollen. On the other hand, Yerlikaya [28] points out that the addition of pollen to a fermented milk beverage has a negative effect on the sensory properties, but taking into account the properties of pollen, it is suggested that after additional technological correction, such as the addition of aromatic ingredients, fruit pulp, or sweetener, this product should be acceptable.
- A significant viability of probiotic cultures was observed in the production of fermented milk beverages supplemented with pollen that are often characterized as functional probiotic products [25,28]. Furthermore, Yerlikaya [28] observed an increase of total solids (from 9.0% ± 0.1% to 10.1% ± 0.1%), proteins (from 3.4% ± 0.1% to 3.6% ± 0.02%), and proteolytic activities (from 0.6 ± 0.28 to 1.5 ± 0.25 expressed as free amino acids equivalents) and a decrease in the content of fat (from 1.6% ± 0.1% to 1.4% ± 0.1%) in the fermented milk beverages supplemented with the commercial bee pollen in a concentration range from 2.5 to 20 mg/mL. Karabagias et al. [24] showed that the total phenolic content and in vitro antioxidant capacity of the yogurts enriched with bee pollen depend on the concentrations of pollen (0.5; 1.0; 2.5; 3.0% *w*/*v*), and they significantly varied in cow (2882.5 ± 1.31 to 7771.5 ± 2.29 mg GAE/L and 71.9% ± 0.02% to 98.79% ± 0.01% inhibition of DPPH radical, respectively), sheep (2900.3 ± 2.25 to 8780 ± 2.25 mg GAE/L and 74.65% ± 0.01% to 99.69% ± 0.01% inhibition of DPPH radical, respectively), and goat (2198.3 ± 1.53 to 7490.5 ± 0.5 mg GAE/L and 71.5% ± 0.01% to 95.91% ± 0.02% inhibition of DPPH radical, respectively) yogurts. Based on the detailed antioxidant and sensory characterization data the “bee pollen yogurts” have been proposed as a new biofunctional food, with the potential for treating chronic human health conditions. However, these hypotheses need to be confirmed by clinical *in vivo* studies [24].

Recently, significant attention has been directed to solid-state fermentation (SSF), which is a very attractive alternative to submerged fermentation (SmF) for the production of several different metabolites in the food industry [39,81]. Solid state fermentation is a specific fermentation process involving microorganisms that can grow on solid materials without the presence of free liquids, while the moisture necessary for the growth of microorganisms is contained and absorbed within a complex and solid matrix structure [82,83]. This process is dependent on several factors, such as type of inoculum, moisture and water activity, pH, temperature, substrate, particle size, and aeration [82]. In addition, Martins et al. [81] emphasized the importance of this process by increasing the content of total polyphenols in food products and utilizing bioactive components from agro-industrial residues. The ideal characteristics of the bee-collected pollen and its ability as a raw material for SSF should be noted, whereby the bee bread product is obtained [84]. It is known that the bees inside the hives prepare and store bee bread, which is produced by the natural bioprocess of bee-collected pollen fermentation [84,85]. Bee bread is a health-oriented product which is characterized by an exceptionally rich chemical composition, nutritional value, digestible properties, and biological activities [5,86]. Hence, in recent years, numerous attempts have been made to imitate the natural bioprocess of the bee-collected pollen fermentation in laboratory conditions in order to develop a suitable product for human consumption [33,34,80,85,86]. However, pollen fermentation is a complex process, primarily due to the morphology of pollen and its solid wall, which requires effective enzymatic activity until the wall is broken and the content is released [87]. In the process of fermentation of the bee-collected pollen, primarily, the species correspond to the genus *Lactobacillus*, whose individual strains possess probiotic properties and the ability to produce bacteriocin [84]. However, Yan et al. [80] showed that the fermentation of pollen by yeasts gives the best potential for obtaining highly valuable nutritional products, due to the substantially increased amounts of components such as amino acids, (from 8.96 ± 0.05 to 44.49 ± 0.15 mg/kg), fatty acids (from 32,184.84 ± 189.24 to 45,427.95 ± 826.47 mg/kg), polyphenols, oligopeptides (from 41.03% to 66.79% of the total determined oligopeptides belong to the peptide molecular weight <1000 Da), and vitamin B class (thiamine, riboflavin, nicotic acid, and nicotinamide). The new protocol for solid state bee-collected pollen fermentation proposed by [34] includes the previously isolated and selected starters which provide a microbiologically stable product with increased digestibility and bioavailability of nutrients, and bioactive constituents present in the initially collected sample of the bee-collected pollen.

### 2.3. Bee-Collected Pollen-Based Bakery, Confectionery, Juice, and Meat Products

The aim of enrichment and incorporation of pollen into foods such as bakery, confectionery, juice, and meat products is to improve the nutritional and functional properties of the final products (Table 2). Nevertheless, due to the complex composition of pollen, its functionality can be highly dependent on the technological processes and the interactions that may occur among the components presented in food and pollen. The properties of food ingredients can directly or indirectly affect the processing, quality, acceptability, and utilization of the product. Since most of the food products are multi-component colloidal systems containing carbohydrates, proteins, lipids, polyphenols, and minerals, and various types of particles, such as oil droplets, gas bubbles, lipid crystals, or starch granules, the properties of products are highly dependent on the nature and the strength of the interactions among these components [88,89]. The influence of pollen’s constituents in foods on physical, thermal, textural, and techno-functional properties, such as solubility, ability to form and stabilize emulsions and foams, and the ability to absorb water and oils, are of great interest during the production process [71,88]. On the other hand, the importance of interactions between the components in the complex food matrix, such as final food products, during digestion, has been extensively investigated in recent years, and it is necessary in order to evaluate the bioavailability and antioxidant activity of integral food components [90,91].

During the processing and storage of some foods, such as meat products, manufacturers often encounter problems, such as uncontrolled oxidation processes, lipid peroxidation, discoloration, and microbial contaminations which affect the quality and acceptability of the products. These problems have been relatively solved by using synthetic additives; however, the toxicity of the active substances of these additives and the increasing consumer demand for healthier products have encouraged the food industry to use natural additives as antimicrobial agents and oxidation inhibitors. Therefore, investigations based on the use of the bee-collected pollen as a supplement in meat products have recently become increasingly interesting to scientists. According to the previous studies, pollen or pollen extract has shown a strong antioxidative effect on the meat products such as sausages or meatballs [45,46,47,92]. A steady increase in TBARS (thiobarbituric acid reactive substances content) was observed during the storage of meat products; however, the addition of pollen significantly slowed down the lipid oxidation compared to the control sample [47]. This is assumed to be due to the gradual release of the bioactive compounds from the pollen, primarily the polyphenols [45]. Additionally, the approximate composition of meatballs formulated with different levels of bee pollen (1.5; 3.0; 4.5; 6.0%) showed and revealed a proportional decrease of moisture (from 57.12% ± 0.24% to 55.00% ± 0.46%) and fat (from 20.65% ± 0.29% to 19.94% ± 0.48%) and an increase of protein (from 16.46% ± 0.08% to 18.94% ± 0.73%) and ash (from 2.19% ± 0.06% to 2.35% ± 0.01%) [46].

The thermal properties of pollen are very important, especially when pollen is used as a supplement in the products that require thermal treatment or roasting at higher temperatures. Under such conditions, decomposition of the nutritional and functional constituents of pollen can occur. Krystyjan et al. [42] suggested that baking biscuits with the addition of pollen (2.5; 5.0; 7.5; 10.0 g), which is a rich source of polyphenolic components (from 1.19 ± 0.11 to 2.31 ± 0.16 mg catechin/g d.m. or from 2.26 ± 0.21 to 4.84 ± 0.23 mg gallic acid/g d.m.), affects their reduction in the final products due to the depolymerization and decarboxylation that can occur during the thermal treatment. Due to the high content of proteins (from 6.96 ± 0.01 to 7.56 ± 0.02 g/100 g d.m.), amino acids, and reducing sugars, baking products to which pollen is added causes the formation of brown pigmented products known as melanoidins [40,42,94]. This finding was confirmed, since significantly higher concentrations of melanoidin were detected in the biscuits enriched with different pollen concentrations than in the control samples [42]. Furthermore, thermal treatment influenced the formation of volatile components such as pyrazine and furan derivatives. Conte et al. [41] examined gluten-free bread enriched with different concentrations of multifloral bee-collected pollen, providing a detailed characterization of the volatile components. It was observed that the addition of pollen potentially influenced the specific reaction pathways involved in the production of some furan derivatives, such as furfural, 2-acetylfuran, and 2-pentilfuran, which are characterized by pleasant sensory characteristics and aromas of caramel, balsamic, cinnamon, fruits, and flowers. To avoid unwanted losses of nutritional and functional properties of the bee-collected pollen, Thakur and Nanda [93] have proposed encapsulation of pollen in milk (carrier) and the development of a new vacuum-dried, bee-collected pollen-rich milk powder that can be successfully applied in the food industry.

It was demonstrated that the botanical origin of pollen was essential for understanding its textural properties, such as hardness, adhesiveness, gumminess, resilience, springiness, cohesiveness, and chewiness [71]. Namely, Thakur and Nanda [71] found that the coconut bee pollen was harder (hardness value was 39.88 ± 6.34 N) than those of the coriander and rapeseed (hardness values were 3.66 ± 1.72 and 15.90 ± 3.14, respectively) due to lower fibre and moisture content. On the other hand, the rapeseed pollen showed the highest adhesiveness (4.10 ± 2.13 g s) compared to the coconut and coriander samples (−4.29 ± 2.71 and −20.81 ± 1.09, respectively), which was attributed to different porosity of the pollen grains. Resilience, springiness, and cohesiveness were the highest in the rapeseed pollen (28.07 ± 5.17, 52.71 ± 4.11, and 0.43 ± 0.05, respectively), but the lowest in the coriander pollen (9.15 ± 3.78, 16.18 ± 2.15, and 0.09 ± 0.02, respectively), which was explained by different chemical compositions, cultivars, and structural integrity. The coconut pollen showed the highest gumminess and chewiness (1527.66 ± 9.43 and 517.71 ± 3.96, respectively), while the coriander pollen had the lowest values (33.70 ± 2.16 and 5.45 ± 1.52, respectively).

According to Conte et al. [40] the increase of pollen concentration in the gluten-free bread significantly improves its textural properties, such as crumb, crust and crumb colour, uniformity of crumb cell, and the structure of crumb grain. Pollen-enriched gluten-free breads showed hardness values significantly lower (<3.18 ± 0.52 N) than the control sample (3.79 ± 0.26 N), while the cohesiveness of crumbs significantly increased in all experimental breads with the addition of the bee-collected pollen to the level of up to 4% [40]. In a similar study, the biscuits supplemented with bee-collected pollen were significantly softer than the control sample, characterized by significantly higher penetration work (from 56.25 ± 3.80 to 79.89 ± 2.30 N·mm) and changes in penetration work during storage, which strongly depended on the presence of pollen [42]. Further, textural profile of meat products such as meatballs formulated with different levels of bee pollen (1.5%; 3.0%; 4.5%; 6.0%) confirmed the influence and proportional decrease of hardness (from 39.18 ± 1.23 to 33.61 ± 0.45 N), springiness (from 0.93 ± 0.01to 0.58 ± 0.03 cm), gumminess (from 6.79 ± 0.42 to 4.97 ± 0.36 N), and chewiness (from 5.93 ± 0.55 to 2.88 ± 0.24 N/cm) with the increase of pollen addition to these products [46].

The solubility of proteins and carbohydrates is a very important techno-functional property, which greatly affects other properties, such as emulsifying, foaming, and gelling [95]. Furthermore, the protein-polysaccharide complex that can be present in a complex food matrix can display better techno-functional properties than proteins and polysaccharides alone due to their highly composite structure obtained through the complex interactions, such as coacervation phenomena, electrostatic and non-electrostatic (hydrogen bonds, hydrophobic interactions and covalent bonds) interactions. Such complexes can be easily modified by processing parameters (pH, temperature, pressure, ionic strength, sharing rate and time, biopolymer charge density, concentration, and ratio), enabling their use as fat replacers, textural agents, and stabilizers [96]. Emulsions play a key role in shaping and forming the structure of food. According to Kostić et al. [88], bee-collected pollen has good emulsifying properties, which gives it the ability to be used in numerous emulsion-based food products. Possessing good emulsifying properties, pollen addition to a natural emulsifier in the production of gluten-free bread can have an effect on reducing crumb hardness [40]. However, Thakur and Nanda [93] showed a strong emulsion activity and stability of the coconut bee-collected pollen and poor emulsion activity and stability of the rapeseed bee-collected pollen, correlating this with the hydrophobicity, conformation, concentration, and solubility of the proteins present in pollen. The observed opposite trend points out, once again, the importance of the botanical origin of pollen.

Unlike the emulsifying properties, according to the report of Kostić et al. [88], bee-collected pollen does not have foaming properties, which reduces its use in products such as toppings, ice-cream, etc., that depend on the incorporation of air to maintain their structure and texture during or after their processing. However, these results are in contrast with the pollen samples from India, which possess high foaming properties and foam stability [93] which could be caused by different methodology and/or different types of pollen applied in research.

Water and oil absorption capacity is a very important feature for the acceptability of the final product. Based on the results obtained, Kostić et al. [88] concluded that the bee-collected pollen had poor water absorption capacity (WAC) and excellent oil absorption capacity (OAC), which is consistent with the results obtained by Thakur and Nanda [93]. The presence of ingredients with high WAC can make food products brittle and dry, especially during storage, while on the other hand, the expressed capacity for oil absorption of any food ingredients is important because lipids act as a flavour retainer and an enhancer of mouth feel [88,95]. According to these facts, pollen has ideal characteristics for use in the formulation of many food products.

## 3. Application of Pollen as a Functional Feed Ingredient

### 3.1. Bee-Collected Pollen as a Feed Source

Like any diet, livestock diets are composed of various components, such as antioxidants, antimicrobials, emulsifiers, vitamins, and minerals to target biochemical and functional needs in poultry. Due to the incidence of diseases leading to a low level of performance, and thus economic losses, antimicrobial protecting agents are incorporated into poultry feed in order to prevent the occurrence of successive microbial maladies. Ensuring a good level of nutritional quality, they are is also obtained through the addition of other feed additives [97]. Ever since the European Union banned the application of antibiotics in 2006, scientists’ attention has been drawn to finding alternative natural feed additives, such as bee-collected pollen [98,99]. To date, researchers have focused on underlying the impact of bee-collected pollen as a positive promotor agent for the growth and fertility of broiler chickens [100,101,102,103,104,105,106]. Biologically, addition of bee-collected pollen to feed portrays its role in the chemical composition profile of broilers; for instance, it increases the water content, resulting in the production of more tender meat. Moreover, pollen can reduce fat content, which in turn, leads to a healthier product and an increase in the protein content of the final products [107]. Bee-collected pollen can also enhance an assortment of blood indices, such as lymphocytes and haemoglobin, and mitigate the negative impacts, such as high cholesterol, creatinine, and uric acid, by reducing said chemicals’ presence in blood [108]. Furthermore, reduction of creatine kinase enzyme was observed in the bee-collected pollen-fed chickens [109]. On top of it all, bee-collected pollen has the potential to improve the immune system, which is likely due to its strong micronutrient profile [110]. Dosage of bee-collected pollen in the diet is considered to be a critical point in the final quality of the produced meat. Bee-collected pollen supplementation in various doses, including 2500, 3500, and 4500 mg/kg, positively affects the chemical composition of the breast meat, increases the water content, and reduces the amount of fat [111]. This addition could also improve the average daily body weight and fertility of chicken broilers during feeding time. Additionally, bee-collected pollen can strengthen the antimicrobial activity along with its micronutrient spectrum which favours the metabolism and health of birds [112]. The dosage of 400 mg/kg bee-collected pollen used in diet could increase the growth performance and body weight in broilers [113]. This is consistent with the recently published report which shows that bee-collected pollen elevates body weight and cumulative feed consumption rate in the broilers fed 1000 mg of bee-collected pollen per 100 kg [114]. Increased body weight might be associated with the specific lipid constituent profile of bee-collected pollen along with a group of trace elements that could modulate the gastrointestinal system and permeabilize the epithelial cells to absorb more nutrients [115]. Haščík and his co-workers [116] conducted a study on the addition of bee-collected pollen at a dosage of 400 mg/kg to the diet of Ross 308 broilers. It was found that bee-collected pollen could have a slight effect on the growth performance, and a 68.5 g increase in body weight of the group fed with the bee-collected pollen was observed, compared to the control group. Prakatur et al. [117] investigated concurrent addition of bee-collected pollen and propolis to the chicken diet; body weight differed significantly on the 7th, 14th, 21th, 28th, 35th, and 42nd days of experiments. They also reported significant differences in the weight gaining rate during the 1st, 2nd, 3rd, 4th, and 5th weeks of the experiments. The increased body weight gaining rate is caused by the bee-collected pollen micronutrients and their role in stimulating the digestive tract of broilers to absorb more nutrient via making the villi thicker and longer [101]. Furthermore, bee-collected pollen contains various enzymes which support the gastrointestinal system [113,116], resulting in higher values for body weight gaining.

There are also studies that suggest a positive influence on the Japanese quails by supplementation with bee-collected pollen [118]. The dosage of 1 g/kg was reported to improve the fatty acid profile (in particular in the case of poly unsaturated fatty acids) in the Japanese quail [119]. Similar results were reported upon addition of the bee-collected pollen to rabbits’ diet: the average daily intake, net profit, and the final body weight of rabbits supplemented with 350 mg/kg of the bee-collected pollen were higher compared to the control group [120]. The blood parameters, such as haemoglobin, urea, and packed cell volume, differed significantly.

### 3.2. Bee-Collected Pollen as a Feed Antioxidant

Since the application of synthetic antioxidants is under scrutiny, owing to its potential toxicity, researchers have averted their attention to the search for natural food sources to meet the consumers’ demands [121]. Several studies have proposed bee-collected pollen as a possible source of natural antioxidants by which lipid oxidation in the meat and meat products could be prohibited [119,122,123]. To evaluate the level of lipid oxidation, malon-dialdehyde (MDA) could be considered as a biomarker that depicts the oxidation level in meat. Bobko et al. [121] studied the impact of bee-collected pollen (at the dosage of 40 mg/kg), and a probiotic (*Lactobacillus fermentum*), and found that MDA amounts in the breast and thigh muscles of treated chickens were 0.127 and 0.119 mg/kg, i.e., 0.128 and 0.139 mg/kg respectively, compared to the control group—0.128 mg/kg in the breast and 0.141 mg/kg in the thigh muscles. The results confirmed antioxidant activity of bee-collected pollen due to lower amounts of MDA in the treated animals. Similarly, Haščík et al. [124] illustrated that the amounts of MDA in the two groups of broilers fed with bee-collected pollen at dosages of 400 mg/kg (1st group) and 800 mg/kg (2nd group) were lower than the control one; they ranged from 0.083 to 0.111 mg/kg and 0.075 to 0.96 mg/kg in the 1st and the 2nd group respectively, compared to the control group, which ranged from 0.105 to 0.137 mg/kg in the thigh meat.

### 3.3. Bee-Collected Pollen and Feed Probiotic

Probiotics are health-benefiting microorganisms located in the gastrointestinal system which manifest their properties when consumed in sufficient amounts [125]. Probiotics can also be useful in the health, nutrition, and growth performance of chickens [126]. These living organisms could induce a positive benefit on the oxidative stability and shelf life of broilers [124], and in particular, they have the potential to be prescribed as proper candidates for antibiotics [127]. Biochemically, the concurrent application of probiotics and bee-collected pollen can help the host’s body develop immunity against resistant microbial strains, thereby improving yield performance in chicken broilers [48]. When the probiotics and the bee-collected pollen are employed together to feed the broilers, this can increase body weight. Adhikari et al. [49] reported that the mixture of probiotics with bee-collected pollen could augment body weight on the 21st, 33rd and 39th days of rising. Furthermore, the most stable thigh meat pH (6.52) is achieved at the storage temperature of −20 °C after 24 h. The combination of probiotics (3.3 g) and bee-collected pollen (at dosage of 400 mg/kg) could improve the fatty acid profile by increasing the essential fatty acids and by reducing the non-essential fatty acids [111].

### 3.4. Bee-Collected Pollen as a Feed Antibiotic

As previously stated, application of antibiotics in the diet of poultry is performed to target growth performance, and at the same time to halt the incidence of microbial diseases [50]. The latter is being nullified due to the risk of spreading antibiotic–resistant strains which inflict high economic costs annually due to treating the infected people [128]. Therefore, to control enteric pathogens which severely affect food safety and health, most of the manufacturers have been advised to provide a diet free of antibiotics [129]. Kačániová et al. [130] investigated the impact of bee-collected pollen on the microbiota of the gastrointestinal system where the lower and the higher count of enterococci were found in the dietary containing 50 and 300 mg/kg of the bee-collected pollen respectively, whilst the lower and the higher counts of *Lactobacilli* were found in the experiments containing 50 g, and 100 and 400 mg/kg of the bee-collected pollen respectively. Application of bee-collected pollen can have an effect on microbiotic availability in the intestinal tract; the higher the doses of bee-collected pollen in the dietary, the lower the production of *Enterobacteriaceae* family in chicken crops [51]. This could be a reason for the bee-collected pollen’s antimicrobial activity [131].

### 3.5. The Impact of Bee-Collected Pollen on Rigor Mortis Stage

In the rigor mortis stage of slaughtered chicken, rapid and deeper reactions were found but with no negative effect on the quality of meat. Also, chickens fed with bee-collected pollen had significant changes of breast colour (being paler) [132]. The results also indicated significant differences between CIE lab parameters (colour space defined by International Commission of Illumination (CIE)) and pHs of the thigh meat and the breast. Furthermore, lower and higher pH might be associated with the poor water binding capacity [133,134] and/or poor shelf life [135]. When broilers were slaughtered, the normal pH ranged from 6.9 to 7.1 at the end of the first hour and 5.7 to 5.9 after 24 h of storage at −20 °C, respectively [49]. Addition of bee-collected pollen (1000 mg/kg) and probiotics (3000 mg/kg) in the feed of experimental broilers increased pH of the thigh meat compared to the control one after 24 h of storage at −20 °C according to Adhikari et al. [49].

## 4. Pollen as a Source of Nutraceuticals

In order to provide new and healthy sources of important food components, human society has developed so-called “functional food” which can be defined as a food prepared in order to afford different compounds (i.e., vitamins, fatty acids, proteins, carbohydrates, polyphenols, carotenoids, etc.) with the ability to have a positive influence on health [136]. If, in that way, functional food helps someone to cure certain disease/disorder, then it can be characterized as “nutraceutical” [136]. Studying of nutraceuticals has increased in last decades due to the significant expansion of cancer all around the world. According to the literature, some of the most important anti-cancer agents naturally present in different foods, are vitamins E and D, polyunsaturated fatty acids (PUFAs), and selenium, the important biogenic trace element [136,137,138,139,140,141]. Special attention among scientific community is given to investigation and usage of plants as a source of polyphenolic compounds. Based on their antioxidant properties, polyphenols originating from different plant sources have been recognized as great anti-cancer agents with confirmed applications in the treatments of skin [142] and colorectal cancer [143]. Pollen as a primary source of nutrients for bees can be used as an excellent source for all above mentioned components in the human diet. Since this aspect of pollen application has been recently well-reviewed several times [4,5,6,9,17], in order to avoid any repetition here, we made brief overview of pollen as a great source of useful and helpful bioactive compounds/nutraceuticals for the preparation of functional foods.

### 4.1. Pollen as a Source of Important Vitamins

Pollen can contain up to 0.7% (0.7 g/100 g) of different lipophilic (vitamins A and E) and hydrophilic vitamins (B group of vitamins and vitamin C) [17]. Alayunt et al. [144] have determined the following ranges of antioxidant vitamins A, E, and C in thirty samples of fresh bee-collected pollen from Turkey: 22.0–53.7 μg/g (vitamin A) 180.0–340.7 μg/g (vitamin E), and 304.3–768.3 μg/g (vitamin C). In addition, six samples of bee-collected pollen obtained from local apiary in Pariquera Açu city (Brasil) contained from 14 to 119 μg/g and 19.4 to 43.0 μg/g of vitamins C and E, respectively [72]. The significant presence of carotenoids, as precursors of vitamin A, is one additional benefit of pollen. These compounds are exclusively related to the tissues included in the process of photosynthesis and can be transferred from plants to animal/human bodies via diet [145]. The average amount of β-carotene in dried pollen is 0.07% (0.07 g/100 g) [17]. It was reported that the amounts of total carotenoids, β-carotene, and provitamin A in fresh Brazilian pollen samples were as follows: 27.1–344.6 μg/g, 3.8–99.3 μg/g, and 0.3–6.5 μg/g, respectively [72]. Similar ranges for the content of total carotenoids (49.9–425.3 μg/g) and β-carotene (0–13.2 μg/g) were determined in fresh Romanian bee-collected pollen samples [9]. Similar to vitamins, carotenoids are quite unstable due to presence of the conjugated double bonds system [145] which is why they are very sensitive hot air drying, extended cooking [145] and storage that is already addressed in Section 2.1.2.

### 4.2. Pollen as a Source of PUFAs

The average content of essential fatty acids (which include PUFAs such as linoleic and γ-linoleic acids) is 0.4% (0.4 g/100 g) [17]. There are several reports about significant presence of PUFAs in pollen samples. For instance, Feás et al. [146] established prevalence of unsaturated fatty acids (55.4%–80.9%) in twenty two samples of bee-collected pollen from Portugal produced as an “organic product.” Among them, PUFAs were represented from 49.6% to 70.8%. Since a “healthy” product should have a ratio of unsaturated and saturated fatty acids (UFA/SFA) higher than 1.6 [147], all examined samples of Portuguese pollen samples had proper values of UFA/SFA ratio (1.9–5.9). Consistently, the range of the content of unsaturated fatty acids in six different floral pollen samples of Serbian maize varieties was from 44.7% to 78.8% (PUFAs range: 22.0%–53.9%) [147]. Finally, in the recent paper about nutritional composition of eight pollen samples collected from apiaries of the Philippine stingless bees [12], it was reported an average UFAs content around 61% (average PUFAs content was 52.2%).

### 4.3. Pollen as a Source of Selenium

The importance of selenium (Se) for the normal functioning of our body is mostly related to its participation as a cofactor in selenoproteins. Namely, this trace element builds the selenocysteine amino acid which is incorporated in the above-mentioned group of proteins, allowing them to contribute in several valuable physiological processes in cells [141]. The average Se content in pollen is about 0.02% (0.02 g/100 g) [17]. Based on the literature data, pollen can be used as a Se source. In that sense, [11] reported that seven floral maize pollen samples contained between 69 and 176 μg/kg of selenium. Three bee-collected pollen samples from Jordan had even more selenium: 1752–3030 μg/kg [148].

### 4.4. Pollen as a Source of Polyphenols

The average content of polyphenols in pollen is around 1.6% (1.6 g/100 g) [17] which defines it as an excellent source of these bioactive compounds. Among said polyphenols content, 1.4% are flavonoids, the most important pollen phenolic compounds [17]. Some authors stated that the content of flavonol glycosides in pollen can be even higher, up to 5%—with the predominant location of these compounds being on the surface of the exine membrane of a pollen grain [13]. The most abundant flavonoids in pollen are quercetin, kaempferol, and isorhamnetin and their derivatives. There are dozens of papers with detailed polyphenolic profiles of different pollen samples from all around the world. For example, the results reported for monofloral pollen samples are listed below:

- Almond (*Prunus amygdalus*) and ‘Jara’ (*Cistus* sp.) floral pollen from Murcia University (USA) contains quercetin, kaempferol, and isorhamnetin and its derivates as the main phenolic compounds [13].
- Rape (*Brassica napus*) bee-collected pollen from Tibet Plateau was characterized as an excellent source of kaempferol (23.4 mg/g) and quercetin (1.4 mg/g) [149].
- Several monofloral bee-collected pollens (*Alternanthera*, *Anadenanthera*, *Myrcia*, *Cocos nucifera*, *Mimosa caesalpiniaefolia*, and *Mimosa scabrella*) samples from Brazil were characterized as a source of different flavonoids (quercetin, naringenin, kaempferol, isorhamnetin, etc.) and flavonoid-3-*O*-glycosides (rutin, isorhamnetin-3-*O*-glycosides, etc.) [150].
- Monofloral bee-collected sunflower pollen (*Helianthus annuus* L.) predominantly contains either quercetin, kaempferol, isorhamnetin and its derivates (sample from Serbia, with total amount ranging between 188.8 mg/kg and 228.2 mg/kg of dry weight) or luteolin, apigenin, and quercetin (sample from Slovakia, in following quantities: 63.6, 32.0, and 10.2 mg/kg, respectively) [15,151].

Additionally, there are several different flavonoid glycosides in pollen which are important as chemotaxonomic markers [13]. But, as nutraceuticals, all polyphenols found in pollen are important anti-cancer agents as already mentioned in introduction part of this section. Also, there is an increasing number of studies showing the importance of flavonoids in the fight against another disease of modern society—Parkinson’s disease (PD). According to recently published review article [152], flavonoids are one of the most prominent agents which can prevent or slow down development of this disease by protecting neurons from oxidative stress. Apart from flavonoids, vitamin C and β-carotene, as compounds frequently present in pollen, can also act as an anti-PD agents [152].

## 5. Anti-Nutritional Properties of Pollen

Apart from richness of different nutrients and useful bioactive compounds, in some cases, pollen can be contaminated with a few compounds which act like anti-nutrients in human body. Unlike nutrients, the list of anti-nutrients in pollen is quite short and can be categorized as follows: allergens, pyrrolizidine alkaloids, toxic and potentially toxic elements, and mycotoxins.

### 5.1. Pollen as an Allergen

Much attention has been paid recently to the study of allergic reactions and their association with foods which are considered to be their main triggers. In addition to classic food allergies involving direct reactions to food allergens (milk, soy, nuts and cereal proteins, egg albumin, etc.) and whose sensitization occurs via the gastrointestinal tract, special attention has been paid to the pollen-related food allergens [52,153]. In this type of allergy, allergic reactions occur as a result of cross-reactions of molecular structures of aeroallergens (inhalant allergen) and food-derived allergens, while primary sensitization occurs via inhalation [52]. Most pollen allergies, such as hay fever (about 10%–25% of population), are related to allergy triggered by air-present pollen, while allergies when pollen is ingested are very rare, similarly to other foods [1]. About 90% of pollen-sensitive individuals show allergic reactions to food that cross-reacts with pollen [154].

Symptoms of allergic reactions can occur within a few minutes to two hours after the ingestion of food, and can affect various organ systems, including the oral mucosa, respiratory tract, cardiovascular systems, and skin. Additionally, special attention should be paid to the more frequent severe allergic reactions, the so-called anaphylactic reactions, which are closely related to food allergies associated with pollen [52]. However, food pollen syndrome is most commonly manifested through characteristic allergic symptoms of itching or burning sensations on the skin, lips, mouth, and throat (acute or contact urticaria of the oropharyngeal sites), when eating certain types of raw fruits and vegetables [153]. The profiles of potentially cross-allergic reactions can be very complex and very broad, and it can be difficult to track individual sensitizations with specific aeroallergens, but the principle of these cross-reactions is based on the binding of IgE antibodies to homologous allergen structures (conformational epitopes) [153,155].

The most common pollen allergens are water-soluble proteins or glycoproteins capable of easily and quickly diffusing after contact between the pollen grain and the mucosa. Additionally, depending on the pH value, time, and temperature [156], pollen grains in the conditions of high humidity and hydration [157] can release allergens or allergen-containing matrices that easily reach the airways (for example, eicosanoid-like substances that cross-react with leukotriene B4 and prostaglandin E2). About 10%–20% of patients with pollen allergies possess strong cross-reactivity with many types of vegetables; e.g., IgE antibodies that are directed against the carbohydrate epitopes of vegetable glycoproteins [158].

Inhalant allergens, such as tree pollen (e.g., birch pollen), have been recognized for their common cross-reactions to foods, while the cross-reactions to foods of *Artemisia*, *Ambrosia*, and grass pollen are still unproven or their cross-reactions with foods are extremely rare [153]. The major birch pollen allergen is defined as Bet v 1 (recognized by more than 95% of patients allergic to birch pollen), which belongs to the PR-10 protein family (pathogenesis-related protein family 10), whose members are dominant allergens for pollen-associated food allergies, while others are less known allergens Bet v 2, Bet v 6, Bet v 7, and Bet v 8 (recognized by 10%–32% of patients allergic to birch pollen) [52,153].

The allergy related to the cross-reaction of olive pollen with food comes from homologous components known as profilin, nsLTP (nonspecific lipid transfer protein; e.g., Ole e 7 allergen associated with severe clinical symptoms), and glucanase [153,159]. Furthermore, fruits such as peaches, apples, pears, kiwi, melons, and nuts are known to cause oral allergy syndrome in patients with an allergy to olive pollen [52]. In the case of *Artemisia vulgaris* pollen (the Art v 1 glycoprotein is recognized as the dominant allergen), the cross-reactions of its allergens with food are rare and insufficiently confirmed, but are known to lead to more severe anaphylactic reactions [153,160].

Grass pollen is one of the most important airborne allergens and its sensitization and cross-reactivity can be attributed and related to specific IgE antibodies that respond to carbohydrates (glycans, especially from plant foods) or to a group of proteins known as profilins [52,161]. The cross-reactions of ragweed-specific allergens with food and their sensitization are still poorly understood. Cross-allergic reactions are often the result of mild allergic symptoms; however, in the event of a large amount of protein (allergen) consumed, serious systematic reactions may occur. For this reason, good information on allergens, their distribution and presence, their characteristics (thermal and pH stability), and their potential danger is required [52].

Allergens, or their protein forms involved in causing allergic reactions, are often subject to modifications during food processing, such as protein unfolding and aggregation, and are present in the complex food structures (which may affect the kinetics of allergen release) [162]. In most pollen-related foods, potential allergenic structures are thermally labile, and the vast majority of potentially allergic people tolerate these foods after heating by boiling, baking, or cooking [52]. Some cases of deviation from this rule have been observed in roasted hazelnuts and cooked celery which may cause allergic symptoms in highly sensitive patients due to the residual and preserved small amounts of pollen-related allergens [52,163], such as lipid transfer proteins in hazelnut Cor a 8 (heat resistant below 100 °C) or celery Api g 2, which show stability during heat treatment [154,160,164,165]. In the studies of Hansen et al. [166] and Worm et al. [163] the complete tolerance to roasted hazelnut was confirmed in 70.6% and 15% of cases, respectively, among the subjects with a confirmed allergy to raw hazelnuts. Similar studies reported by Ballmer-Weber et al. [167] found that 45.5% of the subjects had no allergic symptoms to cooked celery, while Bohle et al. [168] showed no allergic symptoms to cooked celery, carrots, and apples. Also, the observed allergic reactions in subjects to processed foods may not only be due to the presence of thermally stable allergens but may also be closely dependent on the type of pollen sensitization [Lyons et al., 2018]. Furthermore, Verxoeckx et al. [164] suggested that only the fermentation and hydrolysis can affect the potential reduction of allergenicity to the extent that symptoms will not be expressed.

### 5.2. Pyrrolizidine Alkaloids (PAs)

There are several reports about the presence of different pyrrolizidine alkaloids (PAs) in pollen [1,54,169,170]. As secondary metabolites of plants belonging to some genus (*Crotolaria*) or tribes/families (Senecioneae, Eupatorieae, and Boraginaceae), these compounds can be found only in several pollen types [54]. PAs toxicity for humans is related to hepatoxicity and possible developments of lung cancer [53]. According to [1] these compounds can be detected in the pollen samples from Southern Europe (especially due to the presence and use of pollen from *Echium* plants in the Mediterranean countries), while its presence in pollen from the other parts of our continent is not expected. The obtained results for PAs content in pollen samples are extremely different, ranging from 1.08 to 14,000 μg/g for the samples from Germany [54,169,170].

### 5.3. Toxic and Potentially Toxic Elements in Pollen

Mineral composition of pollen is significantly influenced by its botanical and geographical origin [171,172]. Mostly, it is strongly related to growing soil composition or possible anthropogenic influence. In case of toxic and potentially toxic elements, they are frequently the consequence of some contaminations of pollen samples. Among the most represented toxic and potentially toxic elements in pollen are: lead, cadmium, aluminium, strontium, arsenic, mercury, nickel, and chromium. There are several articles which deal with this topic [11,12,56,88,173,174,175]. Based on experience and several studies, Campos et al. [172] made suggestions for allowable contamination levels in pollen samples for: lead (0.5 mg/kg), arsenic (0.5 mg/kg), cadmium (0.1 mg/kg), and mercury (0.03 mg/kg). Currently, there is no recommendation for aluminium, which is one of the potentially toxic elements that can be present in a significant quantity (sometimes more than 100 mg/kg) in pollen. Due to potential neurotoxicity of this element [176] it may be necessary to define a maximal tolerance level for aluminium in pollen, and one for every other toxic element.

### 5.4. Mycotoxins in Pollen

Mycotoxins are secondary metabolites of different fungi from the following genera: *Aspergilus*, *Penicillium,* and *Fusarium*. Over the past few decades, the more frequent occurrence of these fungal products in pollen has been conditioned by adequate pH and *a_w_* values of pollen as fungal substrates and increased temperature and air moisture all around the world caused by climatic changes. Among several different compounds which are defined as mycotoxins, the most dangerous for humans are aflatoxins (AFs) and ochratoxins (OTs)—due to their proved carcinogenic effect. Recently, the presence of mycotoxins in pollen samples has been well-reviewed [55]. According to all collected data, eighteen different mycotoxins were detected and reported in pollen samples collected all around the world with great prevalence of aflatoxin B1 (AFB1), ochratoxin A (OTA), zearalenone (ZEN), and deoxynivalenol (DON) and its derivates and fumonisins (FBs). Authors from several European countries (Slovakia, Serbia, Spain, Portugal, and Greece), and from Brazil, Argentina, and Egypt have reported the following ranges for above mentioned toxic compounds in pollen samples: 0.00–16.20 μg/kg (total AFs), 0.00–17.32 μg/kg (AFB1), 0.00–10.98 μg/kg (OTA), 115.60–361.30 μg/kg (ZEN), 133.30–273.90 μg/kg (DON), 6.30–12.60 μg/kg (total FBs), 113.90–364.90 μg/kg (T-2 toxin), 22–30 μg/kg (neosolaniol), and 1 μg/kg (nivalenol). Currently, there are only suggested limit values for total AFs (4.2 μg/kg) and AFB1 (2 μg/kg) in pollen, proposed by Campos et al. [172]. In the future, the suggestions about maximal allowed concentrations for the other mycotoxins in pollen should be established [55].

## 6. Suggestions and Perspectives for Further Examinations

Application of bee-collected or floral pollen in the formulation of functional food and feed products is in progress. Several important conclusions can be withdrawn: (1) Pollen’s addition to a food matrix generally improves the nutritional, functional, techno-functional, and sensory properties of the newly formulated food products. (2) These improvements depend on the concentration of pollen in the final products. (3) Pollen addition to feed matrix improves the nutritional and functional properties of feed and the quality of slaughtered meet intended for human consumption. (4) The botanical origin of pollen; its chemical composition; the amounts of major bioactive compounds, such as polyphenols and carotenoids; and its antioxidant properties, are not provided in the majority of studies. This should be provided to better understand the impact of pollen addition to food/feed matrix and to standardize the production processes. (5) Special attention should be paid to the quality of applied pollen and the contaminants that can harm human and animal health. Existing regulations should be extended to cover all harmful compounds. (6) Pollen-based food products should be clearly labelled to show the presence of pollen allergens, in order to avoid allergic reactions from pollen-sensitive people.

New technologies such as micro and nano-encapsulation can be used to protect sensitive pollen compounds during processing, storage, and digestion of pollen-based food products, enabling their targeted release and maximum impact on human/animal health. Furthermore, it should be emphasized that the beneficial effects of pollen addition to food matrixes need to be confirmed in in vivo studies.

## Figures and Tables

**Table 1:** Fermented food products based on the application of bee-collected and floral pollen.

Products	Bee-Collected/Floral Pollen Sample(s)	Geographical Origin of Bee-Collected Pollen	Microbial Species Involved in Fermentation	Observations	Reference
**Fermented Pollen-Based Beverages**					
Fermented bee-pollen product with Kombucha/SCOBY consortium	Multifloral bee-collected pollen	Romania	Kombucha/SCOBY (symbiotic culture of bacteria and yeasts) consortium	The aim of this study was to achieve better bioavailability and health effects of bee-collected pollen by fermentation with the Kombuh/SCOBY consortium. The obtained results indicated that the addition of bee-collected pollen increased the proportion of LAB (lactic acid bacteria) in the total number of SCOBY microbial strains, acted as a fermentation activator, increased the content of bioactive compounds (total phenolic and flavonoid content) in the liquid phase of the fermented product which originate from pollen. The obtained product exhibited a moderate antitumor effect on Caco-2 cells.	[19]
Mead (honey-wine) with pollen addition	Commercial bee-collected pollen	Spain	Commercial wine yeast strain of *Saccharomyces cerevisiae*, ENSIS-LE5^R^	Due to the low nutritional content and dilution of honey, problems with the production of fermented honey-wine products such as interfering with or stopping fermentation, poor quality and the undesirable aroma of the product, can occur. In order to avoid these problems, the possibility of addition of pollen in different concentrations (from 0.1 g/L to 20 g/L) as a fermentation activator was estimated. The results showed that the pollen supplementation improved fermentation, honey must composition, and sensory properties. However, it should be noted that the improvement did not correlate with the concentration of pollen.	[20]
White wines with pollen addition	Commercial bee-collected pollen	Spain	Commercial active dry wine yeast strain of *S. cerevisiae* Lalvin 71B^®^	The influence of the addition of pollen (pollen concentration ranged from 0.1 g/L to 20 g/L), as an activator during the alcoholic fermentation of white wines was investigated, with the aim of defining its optimal concentration. Pollen supplementation increased the nitrogen absorption by yeast, provided the maximum of yeast population, and aided the survival of yeast during the death phase. However, doses of pollen higher than 10mg / L showed a significant increase in the volatile acidity, while the optimal recommended activator dose was below 1 g/L.	[21]
White wines with pollen addition	Commercial bee-collected pollen	Spain	Commercial active dry wine yeast strain of *S. cerevisiae* Lalvin 71B^®^	The effect of pollen supplementation on the volatile compounds, the value of odour activity and sensory characteristics in white young wines were investigated, with the aim of defining the optimal concentration of pollen. Pollen supplementation proved to increase the amount of volatile compounds, increase the synthesis of higher alcohols, methanol, esters, acetaldehyde, and terpenes; reduce alcohols, and fatty acids, while at lower concentrations of added pollen, it had a positive effect on sensory and aromatic characteristics, giving a pleasant floral and fruity aromatic profile to the wine.	[22]
Malt beverages enriched with bee-collected pollen	1. Poppy bee-collected pollen (*Papaver somniferum* L.), 2. Rape bee-collected pollen (*Brassica napus* var. *napus*), 3. Sunflower bee-collected pollen (*Heliathus annuus* L.)	/	Brewer’s yeast	The aim of this study was to consider the antioxidant potential (determined by DPPH and reducing power assays), total flavonoids, and polyphenols in sugary beverages when different pollen types were added. Pollen is a rich source of polyphenols that can contribute to the antioxidant potential of the final product such as beer. Test results showed that the pollen-supplemented wort samples had a significantly higher content of polyphenols, flavonoids and antioxidant potential compared to pure wort. Among the investigated samples, highest polyphenolic content was observed in wort with 0.6% of frozen poppy pollen (121.23 ± 2.18 mg GAE/L), while the dominant flavonoid content was in wort with 0.6% of dry poppy pollen (17.20 ± 0.35 mg QE/L). The antioxidant capacity was consistent with the amount of polyphenol content. Therefore, it can be concluded that pollen can be used very successfully in the brewing industry. Particularly prominent are the specimens with the 0.6% addition of bee-collected pollen, mainly the pollen of *Papaver somniferum* L.	[23]
**Fermented Pollen-Based Dairy Products Industry with the Addition of Pollen**					
Bio-functional, “bee-collected pollen yogurt “	Commercial bee-collected pollen originating from a mixture of flowers	Greece	*Streptococcus thermophilus*; *Lactobacillus bulgaricus*	Preparation of cow, sheep and goat milk yogurts with different pollen concentrations were studied in terms of antioxidant capacity, total polyphenolic content, and sensory evaluation. According to the results, pollen-enriched yogurts showed a significantly higher antioxidant capacity and polyphenolic content compared to conventional yogurts. Furthermore, taste, odour, appearance, and cohesion were significantly improved, while the bee-collected pollen yogurts of 0.5%–1.0% (*w*/*v*) were proposed to be the new bio-functional foods.	[24]
Bio-yogurt with probiotic bacteria, royal jelly and bee-collected pollen grains	Bee pollen grains from the apiary of the Faculty of Agriculture, Moshtohor, Benha University, Toukh, Kaliobia	Egypt	**Yogurt starter cultures:** *L. delbrueckii* spp. *bulgaricus* *S. thermophilus* **Probiotic bacteria:** *Bifidobacterium angulatum* DSM 20098; *Lactobacillus gasseri* ATCC 33323; *Lactobacillus rhamnosus* DSM 20245	This study aimed to produce a bio-yogurt with probiotic bacteria and with the addition of royal jelly and bee-collected pollen grains, and to evaluate the functionality of the products, their sensory properties and microbial stability during storage. The results showed that the addition of probiotics, royal jelly and bee-collected pollen to the yogurt increased the coagulation time and nutritional quality, and improved the sensory and rheological properties of the produced functional yogurts compared to the control ones.	[25]
Probiotic yogurt supplemented with the bee-collected pollen grains	Bee pollen grains from the apiary of the Faculty of Agriculture, Moshtohor, Benha University, Toukh, Kaliobia	Egypt	**Yogurt starter cultures:***L. Lactobacillus delbrueckii* spp. *bulgaricus* *S. thermophiles* **Probiotic bacteria:** *B. angulatum* DSM 20098; *L. rhamnosus* DSM 20245; *Lactobacillus gasseri* ATCC 33323	The aim of this study was to investigate the effect of bee-collected pollen supplementation on the texture, microstructure and stability of the preserved probiotic yogurts. Based on the obtained results, pollen-added probiotic yogurts were more stable during the cold storage for 21 days. Pollen supplementation did not significantly affect the textural properties of the yogurts such as cohesiveness and springiness compared to the control sample, but springiness, gumminess and chewiness were increased during the yogurt storage. Highest hardness (1.96 ± 0.02 N) was observed in the yogurt T5 (1.5% yogurt starter + 1.5% *Lb. gasseri* + 0.8% bee pollen grains). Furthermore, significantly decreased syneresis was observed compared to the control sample. The addition of pollen influenced the microstructure of the yogurt and the formation of a more comprehensive network, which contributed to improved consistency and water retention capacity.	[26]
Yogurt with bee-collected pollen supplementation	Commercial bee-collected pollen	Bulgaria	*S. thermophilus*; *L. delbrueckii* ssp. *bulgaricus*	According to the established technological production procedure (Bulgarian producers that use technology according to BNS 12:2010), yogurts were made with and without the addition of honey and bee-collected pollen (0.4; 0.6; 0.8%), in order to define the main physicochemical and organoleptic parameters. Based on the obtained results, it was found that the addition of honey up to 5% and pollen up to 0.4% improved the organoleptic and physicochemical properties of the final products. However, it can be observed that the content of vitamin C in the yogurt samples increased (from 0.45 to 0.95 mg/100 g) with the increase in the amount of pollen.	[27]
Fermented milk beverages with bee-collected pollen supplementation	Commercial bee-collected pollen	Turkey	Commercial ABT1 starter culture: *Lactobacillus acidophilus* La 5, *Bifidobacterium animalis* subs. *lactis* Bb 12, *S. thermophilus*	The aim of this study was to determine the optimal bee-collected pollen concentration for the enrichment of fermented milk beverages, with the additional evaluation of antimicrobial, chemical, rheological, and sensory properties, and the probiotic viability. The addition of bee-collected pollen affected the acidification profile, showed a positive effect on probiotic viability, partial antimicrobial activity was observed, while the sensory properties were deteriorated requiring additional technological processing to make the product development and usage possible.	[28]
Yogurt supplemented with bee pollen	Three unifloral bee-collected pollens: 1. maize (*Zea mays*), 2. clover (*Trifolium alexandrinum*), 3. date palm (*Phoenix dactylifera*)	Egypt	Starter yogurt culture	This study aimed to evaluate the antibacterial and antioxidant activity of the bee-collected pollen extracts, and the tendency to develop a new pollen supplemented product. Based on the obtained results, all pollen samples showed antibacterial and antioxidant activity, and the ability to inhibit lipid peroxidation, however, the extracts of the maize bee-collected pollen were particularly prominent. The addition of pollen in yogurt production did not affect the starter culture, while the prepared yogurts showed specific sensory characteristics depending on the type of pollen: the maize bee pollen supplementation gave a nutty flavour; the clover bee-collected pollen gave a sweet taste, while the date palm bee-collected pollen gave a beany flavour. In addition, the maize bee-collected pollen improved the texture, increased the gel strength and decreased the syneresis of the yogurt.	[29]
Cheese enriched with bee-collected pollen	Bee collected pollen	Damanhour district, Egypt	*Lactobacillus delbruecki* ssp. *bulgaricus*, *S. thermophilus*	The aim of this study was to investigate the effect of the bee-collected pollen supplementation (0.5; 1.0; 1.5; 2.0%) on the antibacterial, sensory, and physicochemical properties, and the antioxidant activity of white cheeses produced from a mixture of cow and camel milk. Based on the obtained results, the incorporation of bee-collected pollen into cheeses contributed to the significant presence of polyphenols (total polyphenol content was in the range from 11.53 to 46.78 mg/g chesses, depending on the storage period increased the fat and protein content, and increased antioxidant activity (inhibition of ascorbate auto-oxidation, from 1157 to 5261 µg/g chesses, and reducing power assay, from 30.83 to 98.37% inhibition), while the effects on the sensory properties were not observed, compared to the control sample with up to 1% of added bee-collected pollen. Furthermore, the results showed an antibacterial effect on *St. Aureus* (ATCC 6538), *S. typhimurium* (ACTH 25, 566) and *E. coli* (ACC. 8739).	[30]
**Pollen as Fermentation Feedstock**					
Pine pollen fermentation product	Floral pine pollen	China	*Lactobacillus paracasei* Lc-3	The basis of this experiment was a statistical experimental design strategy to optimize the fermentation conditions of pine pollen using *L. paracasei* Lc-3. Therefore, the goal was to develop a product based on the pine pollen with an improved nutritional value.	[31]
Pine pollen fermentation product	Floral pine pollen	China	Isolated and characterized strain of *Bacillus coagulans*	The basis of this experiment was a statistical experimental design strategy, optimized by using Box-Behnken design and response surface methodology, to define the best fermentation conditions of the pine pollen by using the isolated *B. coagulans* strain. Additionally, a single factor experiment was conducted as a preliminary study for the treatment of fermented products which was carried out through spray-drying.	[32]
Bee bread	5 samples of bee-collected pollen	Lithuania; Latvia	1. with *L. rhamnosus* GG (ATCC 53,103) 2. without selected strains	This study represents an attempt to produce bee bread under improvised laboratory conditions and it aimed to evaluate the effect of solid phase fermentations on the total phenolic content, total flavonoid content, and radical scavenging activity of the bee-collected pollen samples. The results showed that, generally, fermentation (with and without the use of selected bacteria strains) had a positive effect on the total flavonoid content and the obtained values were comparable to those obtained for natural bee bread.	[33]
Bee bread	1 sample, ivy (*Hedera helix* L.) bee-collected pollen	Italy	mixed inoculum of selected *Lactobacillus kunkeei* strains and *Hanseniaspora uvarum* AN8Y27B	The new proposed biotechnology protocol for the bee-collected pollen fermentation, which involved the use of selected strains of microorganisms, mimicking the spontaneous fermentation of bee bread, confirmed the production of microbiologically stable and safely fermented products with improved nutritional and functional characteristics.	[34]
Fermented bee-collected pollen products	1 sample of monofloral (*Brassica campestris* L.) bee-collected pollen	China	1. LAB (*L. bulgaricus* and *S. thermophiles*) 2. Active dry yeast 3. mix of LAB and yeast	Bee-collected pollen was fermented by various microorganisms, such as, selected lactic acid bacteria (LAB), yeasts, and mixed microbes, with the aim of improving its nutritional properties. The results showed that bee-collected pollen fermented with yeast provided the best characteristics of the product with significantly increased polyphenol, fatty acid, oligopeptide, and vitamin content.	[80]
Fermented pollen cans	Bee-collected pollen sample (*Brassica napus*)	Slovakia	/—Without selected strains	In order to overcome the problem of restricted digestibility of pollen, one of the possible solutions is the production of cans of fermented pollen according to the pattern of the bee bread production by bees. Therefore, the aim of this study was to investigate the different variants of pollen production in cans (for their production fresh bee-collected pollen, honey, boiled water, and yogurt were used) from the point of view of occurrence of the filamentous microscopic fungi (FMFs). The results showed that the number of the FMFs was reduced by fermentation.	[35]
Fermented bee-collected pollen products (‘probiotic characterized product’)	Commercial bee-collected pollen	Colombia	Four different commercial probiotic cultures: 1. *L. acidophilus* 2.*Lactobacillus paracasei* 3. two mixed cultures: (YOMIX^TM^205LYO and CHOOZIT^TM^MY800)	Due to the poor bioavailability of pollen nutrients, an experiment was set up to discuss the fermentation process of the bee-collected pollen which was previously heated in order to obtain a product with good characteristics suitable for human consumption. The results showed that it was possible to produce a fermented product safe for human consumption with a high number of acid lactic bacteria (ALB) viable cells and increased lactic acid concentration, which gave it a probiotic character.	[36]
Pollen-Based Probiotic Product	Botanical origin of pollen was not provided	/	*Lactobacillus* strain (*L. acidophilus*)	This study aimed to identify *Lactobacillus acidophilus* strains used for inoculation in the ground or unground pollen and honey medium to produce a pollen-based probiotic product.	[38]
Functional product based on probiotic biomass, pollen and honey	Botanical origin of pollen was not provided	/	*Lactobacilus* (four strains) and *Bifidubacterium* (two strains)	This study examined the impact of prebiotics, such as inulin and lactulose, on the multiplication of some defined probiotic strains used in pollen and honey in order to produce a product similar to bee bread. The results highlighted a medium based on the ground pollen and honey supplemented with inulin as the best one. After seven days of fermentation, viability was over 300 CFU × 10^6^/g and the total antioxidant activity was over 45%.	[37]

**Table 2:** Bee-collected pollen-based bakery, confectionery, juice, and meat products.

Products	Bee-Collected Pollen Sample(s)	Geographical Origin of the Bee-Collected Pollen	Observations	Reference
Biscuits with bee-collected pollen supplementation	Bee pollen was collected from the beehives located in the south-eastern regions of Poland	Poland	Considering the fact that biscuits are among the most popular and desirable sweet foods, the aim of this research was to develop the optimal recipe for the production of biscuits with added bee-collected pollen, with accompanying physical, chemical, and sensory characterization. The results showed that the addition of bee-collected pollen significantly increased the content of sugars, proteins, ash, fibers, and polyphenols, and the antioxidant potential of the final products, while it had no effect on their lipid content. The addition of pollen affected penetration work, product colour intent, and sensory characteristics (depending on the concentration of added pollen).	[42]
Cookies enriched with rape bee-collected pollen	Rape (*Brassica napus* var. *napus)* bee-collected pollen	Slovakia	The aim of this research was to develop a new product (permanent pollen-added cookie), optimize the concentration of added pollen originating from different localities, and to evaluate the technological and chemical parameters of the final products, that is, sensory characterization. According to the technological procedure, 16 and 32% of wheat flour was replaced by the bee-collected pollen from two localities. The results showed that the pollen addition, depending on its concentration and origin, increased the content of the reducing sugars, protein and ash content, and the antioxidant activity of the final product. Technological parameters such as the diameter and the weight of the cookies were increased, while the thickness of the product decreased with the gradual addition of pollen. The cookies were characterized by pleasant and easy chewiness, with a delicate taste.	[43]
Gluten-free bread enriched with bee-collected pollen	Multi-floral dry bee-collected pollen	Italy	The aim of this study was the addition of different concentrations of pollen in the production of the gluten-free bread, comparison with the control bread sample, and the additional assessment of the physicochemical, technological, and sensory properties of the developed products. Based on the results, it could be observed that the pollen supplement did not affect the rheological properties. On the other hand, the pollen supplement significantly improved the technological characteristics, sensory characteristics, and product acceptability. The bread enriched with 3% bee-collected pollen stood out, especially since it achieved the ideal balance between all the characteristics.	[40]
Gluten-free bread enriched with bee-collected pollen	Multi-floral dry bee-collected pollen	Italy	The aim of this study was to define the nutritional properties, aromatic profile and antioxidant activity of the gluten-free bread with the addition of different concentrations of multi-floral pollen. Pollen-enriched breads showed higher levels of protein, ash, K, Ca, polyphenols, and carotenoids, while there were no observed effects on the lipid content. In addition, the antioxidant activity, bioavailability of polyphenols, and the content of some furans, which are characterized by pleasant aromas, increased.	[41]
Bee-pollen-based beverage (fruit juice with the addition of bee-collected pollen)	Bee-pollen was provided by manufacturers from the Cundiboyacense Highland region	Colombia	The authors intended to optimize high pressure processing (HPP) treatments as a tool for inactivation of microorganisms in order to increase the extractability and the availability of the pollen bioactive compounds. However, a particular area of interest was the impact of the treatment on the pineapple juice-based beverage matrix to which pollen was added. According to the results, HPP treatment improved the extractability of some bioactive compounds present in the bee-collected pollen grains. Therefore, an increase in the total carotenoid content (TCC) (from 43.19 ± 1.19 to 86.60 ± 0.35 mg β-carotene/kg), the total phenolic content (TPC) (from 8.76 ± 0.38 to 20.34 ± 1.08 mg GAE/g), and the antioxidant capacity (FRAP) (from 57.70 ± 2.3 to 140.3 ± 4.9 µmol Trolox/g) in the bee-collected pollen-based beverage was confirmed as a consequence of the HPP treatments. The optimum defined treatment conditions were: pressure 315 MPa, applied for 14.5 min combined with 8% (*v*/*v*) of bee-collected pollen. Also, the treatments showed the effectiveness of the inactivation of microorganisms.	[44]
Bee-collected pollen-rich milk powder	10 samples of bee-collected pollen, unifloral rapeseed (*Brassica napus*) bee-collected pollen	India	This study aimed to develop a vacuum-dried bee-collected pollen-rich milk powder. For the purposes of the process, optimization and the definition of optimal parameters, a response surface methodology based on the results determined for physicochemical and functional properties was applied. The optimized parameters for the production of this functional additive were: 13.71% pollen, 26.84 °C temperature and 23.37 Hg pressure. In addition, characterization of the optimized powder by defining its morphological properties and particle size distribution was carried out. The resulting powder proved to be a significant source of polyphenols, giving it the ability to be used in various industries to make healthier food products.	[93]
Meatballs formulated with bee-collected pollen	Bee pollen was obtained from Fanus Gida ve Organik Urunler San. Tic. Ltd. Sti.,Turkey	Turkey	In this study, the nutritional quality of meatballs with the addition of different amounts of pollen was monitored during production and storage. According to the results, pollen supplementation led to an increase in the content of proteins and polyunsaturated fatty acids, while, on the other hand, a decrease in moisture content and textural changes occurred, primarily a decrease in the hardness and stickiness of the meatballs. Pollen supplementation inhibited lipid oxidation and inhibited bacterial growth in the meatballs. It can be concluded that the addition of bee-collected pollen influenced the nutritional and storage quality of the meatballs with minimal changes in the composition and sensory properties.	[46]
Meatballs formulated with bee-collected pollen	Bee pollen was obtained from Fanus Gida ve Organik Urunler San. Tic. Ltd. Sti.,Turkey	Turkey	It is known that freezing foodstuffs prevents the growth of microorganisms; however, the processes of lipid peroxidation and discoloration have not been completely stopped. Therefore, this study aimed to define how the addition of different pollen concentrations affected the colour and lipid oxidation of the meat balls during storage in a freezer, and microbial quality of the final products. The results showed that pollen addition and retention period affected the colour and pH of the products, while the content of thiobarbituric acid reactive substances (TBARS) in the samples increased during storage, but the addition of pollen slowed down the lipid oxidation. It should also be mentioned that the addition of pollen inhibited the growth of microbes in the meatballs. Based on the results, it was concluded that pollen can be successfully used as a natural antioxidant and antimicrobial agent in meatballs.	[47]
Pork sausages with the addition of lyophilized bee-collected pollen extract	Heterofloral pollen: Arecaceae and Brassicaceae families, *Baccharis* and *Eupatorium* (genus from Asteraceae family)	Brazil	Due to the high polyphenol content and the antioxidant potential of pollen that was previously determined, this study aimed to utilize the lyophilized bee-collected pollen (LBP) extract in the production of pork sausages, from the aspect of preventing lipid oxidation of the product during storage. Sausages with the LBP supplementation paired with low storage temperature showed lower TBARS values during storage compared to the control sample and the sausage sample prepared with sodium erythorbate (SE). It can be concluded that this methodology of sausage production with the addition of polyphenol-rich pollen extract is very effective in stopping the lipid oxidation.	[45]
Black pudding with the addition of bee-collected pollen and bee-collected pollen extract	Predominant pollen grains in bee-collected pollen originating from *Cistus ladanifer*	Portugal	The aim of this study was to characterize bee-collected pollen, which was further used as an additive in the production of black pudding. In addition, microbial quality, water activity, pH, and lipid oxidation of the product were evaluated during four storage periods. The results showed a slight deviation in humidity and pH, the absence of microorganisms, and positive influence on the prevention of lipid oxidation. Accordingly, the authors concluded that the use of pollen as an antioxidant in the formulation of a product improved its quality and acceptability without altering its distinctive traditional taste.	[92]
